# DRIVERS AND RESTRAINERS OF CAPABLE DISSEMINATION

**DOI:** 10.1093/geroni/igac059.1296

**Published:** 2022-12-20

**Authors:** Sarah Szanton, Deborah Paone

**Affiliations:** Johns Hopkins University School of Nursing, Baltimore, Maryland, United States; Johns Hopkins University, Baltimore, Maryland, United States

## Abstract

Using the technique of Force Field Analysis, the co-developer of CAPABLE examined drivers and restrainers of dissemination for the CAPABLE program. This generated 19 distinct drivers including robust research findings, demonstrated clinical and economic utility, and high value to older adults. The environmental shift toward value-based approaches in Medicare and Medicaid also increased the perceived value of CAPABLE. In addition, there were 8 restrainers, including component complexity, siloed health and housing sectors, and the operational lift needed to build the program. Lessons are offered from this case study to move from research to embedded practice.

